# Determining how individuals manage their driving anxiety

**DOI:** 10.1371/journal.pmen.0000163

**Published:** 2025-04-16

**Authors:** Jacob Greenfield, Jadyn Allen, Toni Marie Rudisill

**Affiliations:** 1 Division of Occupational Therapy, West Virginia University, Morgantown, West Virginia, United States of America; 2 Department of Epidemiology and Biostatistics, West Virginia University, Morgantown, West Virginia, United States of America; PLOS: Public Library of Science, UNITED KINGDOM OF GREAT BRITAIN AND NORTHERN IRELAND

## Abstract

Anxiety caused by driving (e.g., driving anxiety) can greatly impact individuals’ mobility and lead to partial or complete driving cessation. Previous research focused on the causes of driving anxiety and quantifying its severity. No studies have centered on how individuals manage their symptoms. The purpose of this study was to learn how and what management strategies individuals utilize to combat anxiety triggers while driving. Semi-structured interviews were conducted with individuals from Jan-March 2024. Participants had to be ≥18 years of age at time of study, reside in the United States, and experienced or were concerned about their driving anxiety. Participants were recruited through email and advertisements using non-probability sampling techniques. The interviews were conducted using a standardized, pilot tested script and were audio recorded and transcribed. A thematic analysis with inductive coding was performed to determine themes. Ten female participants (mean age 40.8 years) were recruited. Five themes emerged: past trauma, 2) environmental factors and their inability to control them, 3) within and out-of-vehicle coping strategies (subthemes: distraction, reliance on others, and avoidance), 4) out of control in unfamiliar situations or fear of hurting self, others, or property, and 5) honoring responsibilities, being independent and maintaining relationships. While management strategies were individualistic, most participants attempted to distract themselves from their feelings, relied on others to deal with their symptoms, or avoided situations that aggravated their anxiety. These management strategies enabled participants to continue driving in spite of their symptoms and allowed them to honor commitments, maintain independence, and sustain relationships. However, these strategies could also inadvertently impact individuals’ safety while driving and/or their mental health. These findings could inform clinical practice and future research.

## Introduction

Driving is an occupation that affords individuals freedom and independence with community navigation; however, this sense of freedom and independence can be significantly altered when individuals experience driving anxiety. In 2022, there were approximately 40 million Americans diagnosed with anxiety, which included Generalized Anxiety Disorder, Panic Disorder, Social Anxiety, and specific phobias, such as driving anxiety [1]. With such a high prevalence of anxiety within the population, it is important to note that driving phobias are one of the most commonly reported phobias [2]. This phobia or anxiety symptoms are most commonly reported in women between the ages of 30–40; however, evidence indicates that men are more likely to experience driving phobias following a motor vehicle collision [2]. Driving anxiety can manifest in various ways, from concern over specific driving conditions of general driving, such as in climate, weather, darkness, or traffic [3], developing into a phobia as the driver or passenger, or may lead to avoidance behaviors [4].

Individuals who experience driving anxiety have reported panic attacks, fear of being judged by others or being involved in a motor vehicle collision as the root cause of their anxiety symptoms [5,6]. Being involved in a motor vehicle collision can be a traumatic experience for many individuals. According to the Post-traumatic Growth Model, these traumatic events have the possibility of promoting personal growth, potentially leading to developing new personal strengths or leading to new possibilities [7].

Individual symptoms such as panic attacks, fear of being judged by others, or being in a motor vehicle collision have been supported throughout the literature as the bulk of research related to driving anxiety revolves around determining the cause of anxiety symptoms [3], and/or utilized a driving anxiety scale to quantify and determine the individuals’ severity of anxiety symptoms. Studies ranging from 1994 through 2020 completed similar procedures to better understand the cause and severity of an individual’s anxiety symptoms [5,8,9] and determined that the majority of participants reported being involved in a potential collision was an anxiety trigger. From these studies, prior motor vehicle collisions were the most common anxiety trigger, followed by the onset of panic attacks due to road conditions [10].

Driving anxiety has also been shown to lead to partial or total avoidance [11]. While avoiding an activity that is anxiety-provoking, avoidance has significant implications on one’s daily life [12]. Employment, social relationships, and health maintenance are typically dependent on daily driving, but avoiding driving can make it difficult to carry out these activities [4]. Avoidance can also be detrimental to an individual’s life if there are little or no public transportation options in their community [13].

While the majority of available literature researching driving anxiety revolves around determining triggers, performance behaviors, and severity, there is little research understanding of how individual’s manage their anxiety symptoms while driving. Additionally, the bulk of studies use quantitative severity or impact scales to determine the overall impact on an individual’s life; additionally, very few qualitative studies could be located in the peer-reviewed literature concerning driving anxiety. Therefore, the purpose of this study was to determine what management strategies individuals utilize to combat anxiety triggers while driving using qualitative methodologies.

## Methods

### Study design and population

To meet the study’s objectives, a qualitative study was conducted, which consisted of semi-structured interviews. The study population included any individual who was 1) ≥18 years of age at time of study, 2) resided in the United States, 3) and experienced anxiety while driving or was concerned about their anxiety while driving.

### Study location and recruitment

Study participants were recruited from January-March 2024. Because this study was phenomenological in nature and qualitative studies are not intended to be generalizable, convenience and snow-ball sampling techniques were employed [14,15]. Study advertisements were posted throughout West Virginia University’s main campus, which is located in Morgantown, WV. Mass emails were also sent to faculty and staff, particularly those in human performance. West Virginia University is located in north-central West Virginia, approximately 1.5 hours south of Pittsburgh, PA. Morgantown, WV is a small city with ~30,000 full-time residents. As of fall 2023, >26,000 students were enrolled with ~3,000 full and part-time faculty employed [16].

### Data collection

#### Interview guide creation and theoretical framework.

An interview script was carefully created prior to conducting the semi-structured interviews. The Health Belief Model (HBM) principles supported the theoretical framework and creation of the interview script to better understand an individual’s specific triggers and management strategies related to driving anxiety. The HBM suggests that a person’s belief in a threat and health behavior/actions predict the likelihood of adopting a behavior [17]. The HBM comprises factors influencing an individual’s behavior: perceived susceptibility, perceived severity, perceived benefits, cues to action, and self-efficacy. The tenets of the HBM were utilized when constructing the qualitative interview script for this study. The questions posed were open-ended and allowed for probing, follow-up questions, and clarification if needed. The interview script was pilot-tested with one individual who experienced driving anxiety.

#### Interviews.

All interviews were scheduled at a convenient time and modality for participants. Participants could participate in an interview on-site, over the phone, or via Zoom conferencing technology. One individual, who was trained in proper interviewing techniques, conducted all the interviews to minimize interviewer bias. All interviews were conducted in a private, quiet location to ensure participant confidentiality. The interviews lasted approximately 20–40 minutes in duration and were audio recorded with the participants’ consent. The audio recordings obtained from the interviews were transcribed verbatim by a professional transcription service. All transcripts were de-identified and checked for accuracy by the authors. Any discrepancies between the audio files and transcript were corrected prior to data analysis. Interviews were conducted until saturation was achieved; this meant that no additional ideas or information relating to driving anxiety and coping strategies were revealed by participants. All authors agreed that saturation was reached after the ninth interview. However, a total of ten interviews were conducted.

Immediately before an interview was conducted, an anonymous electronic survey was administered to the participants using RedCap software. The participant could take the survey using a link and/or QR code on their phone or personal computer. This survey collected basic demographic information and driving history from participants such as age, gender, years of driving experience, miles driven per week, etc. The questions asked on the survey were taken from valid and reliable national health and transportation surveys.

### Data analysis

A thematic analysis was undertaken by the study authors to identify emergent themes; an inductive coding approach was utilized for this process [18]. All authors read and reviewed each transcript independently. All authors met to compare codes that labeled thoughts, ideas, concepts, etc. in the transcripts. A codebook was initially developed and refined over the data analysis process. After several rounds of coding and subsequent meetings among the authors, a thematic map was constructed, and the codes were collapsed into overarching themes. Once the final themes were determined, all authors re-read the transcripts to ensure that the themes accurately represented the data. Data management was conducted using both NVivo Release 1.0 and Microsoft Excel. Demographic data were summarized via frequency and percentages using Microsoft Excel.

## Results

### Quantitative

A total of 10 (n=10) participants completed the interview until saturation was reached. The average age of the participants was 40.8 years, and the average age when their license was obtained was 17.9 years. All participants identified as female and held a current driver’s license. Of the 10 participants, 9 indicated that they currently experienced feelings of anxiety when driving, and 7 participants were able to indicate the cause of their symptoms from witnessing a major accident. The participants reported that they drove during all hours of the day, and the majority (e.g., 80%) drove under 100 miles per week. Driving occurred for social purposes (100%), 80% for work purposes, and 50% for community purposes. Out of the 10 participants, 7 have received an anxiety disorder diagnosis. Participants indicated various forms of symptom management, such as 4 indicated they utilized talk therapy or counseling to manage their anxiety, 2 utilized aromatherapy, and 2 participants utilized meditation. Each participant, either on their initial intake form or during the interview, indicated they had tried or are currently engaged in regular exercise, sleep hygiene practices, or acupuncture to manage anxiety. In addition to non-pharmacological interventions, 6 participants indicated that they used prescribed medication to manage their anxiety (Table 1).

**Table 1:** Participant demographics.

Average Age	40.8	
Characteristic	n	**%**
Gender		
Female	10	100
Male	0	0
Transgender Female	0	0
Transgender Male	0	0
Nonbinary	0	0
Genderqueer/Genderfluid	0	0
Driver’s license status		
Current	10	100
Expired, canceled, or revoked	0	0
Age of initial licensure		
16-20	8	80
21 or older	2	20
Experience with/witness of major accident		
Yes	7	70
No	2	30
Not reported	1	10
Experience symptoms of anxiety while driving		
Yes	9	90
No	1	10
Diagnosed with an anxiety disorder		
Yes	7	70
No	3	30
Previously/currently prescribed anxiety medication		
Yes	6	60
No	4	40
Alternative methods for treatment/symptom relief		
None	3	30
Talk therapy	2	20
Counseling or therapy	2	20
Aromatherapy, medication, acupuncture	1	10
Talk therapy, meditation aromatherapy, and exercise	1	10
Sleep and talking	1	10
Driving time of day		
Morning and evening	3	30
Morning and afternoon	2	20
Daytime	2	20
Morning, afternoon, some evening/night	1	10
Various, daylight hours always, avoid rush hours	1	10
Evening/night (p.m.)	1	10
Current purposes for driving		
Social, work, and community	3	30
Social and work	3	30
Social, work, domestic, and community	1	10
Social, domestic, and community	1	10
Social, work, and domestic	1	10
Social	1	10
Miles driven per week		
Less than 100 miles	8	80
100–300 miles	2	20
300–500 miles	0	0
More than 500 miles	0	0

Note. Responses combined based on participant reports

### Qualitative

Based on the inductive thematic analysis, the following five themes were determined by the research team: 1) *Past trauma, 2) Environmental factors that cause anxiety and the inability to control them, 3) Both out of vehicle and within vehicle coping strategies (sub-themes: distraction, reliance on others, and avoidance), 4) Being out of control in an unfamiliar situation or fear of hurting self, others, or property, and 5) Honoring responsibilities, being independent, and maintaining relationships* (Table 2)*.*

**Table 2:** Core themes identified among participants.

Theme	Description of theme
Past trauma	Any kind of incident or traumatic event that may have triggered the participants’ anxiety. It could have been a traumatic event that happened to them or even someone they knew.
Environmental factors that cause anxiety and the inability to control them	This theme refers to anything that is permanent or semi-permanent in the environment that may cause a participant to feel anxious while driving. This included inclement weather, traffic patterns, road layout, bridges, tunnels, physical signs, time of day, other drivers, and those drivers’ behavior. Many participants felt these were things they could not necessarily control.
Both out of vehicle and within vehicle coping strategies (subthemes: distraction, reliance on others, and avoidance)	This theme included anything or action that a participant would do either when in or out of the vehicle in an attempt to manage their anxiety. Three sub-themes emerged and included distraction, reliance on others, or simply avoiding driving or an aspect of it that made them anxious. When in the vehicle, many participants tried to distract themselves from their anxious thoughts by engaging in activities such as gum chewing, praying, positive self-talk, stopping any distractions, or purposely engaging with distractions such as radio, podcasts, people, etc. Many participants prepared themselves to drive or while driving as a precaution or safety measure. This included route planning, wearing safety belts, driving a bigger car, etc. Some participants relied on others when they were anxious, such as letting someone else drive or calling/talking with a person or passenger. Also, some participants just avoided driving in general or things that made them nervous during their drive as a means of coping. Outside of the vehicle, many participants engaged in anxiety-reducing behaviors such as pharmaceutical, therapeutic, and/or lifestyle changes
Being out of control in an unfamiliar situation or fear of hurting self, others, or property	This theme refers to times when a participant claimed to be afraid of getting hurt, dying, killing someone or something else (e.g., pet), or destroying something such as a car when they were driving. Many participants were also afraid of unfamiliar driving environments and situations where they could get lost and/or not know what to do.
Honoring responsibilities, being independent, and maintaining relationships	This theme refers to personal motivators for someone to continue driving. This often included driving for things such as work (e.g., responsibilities), maintaining autonomy or self-sufficiency (e.g., being independent), or maintaining a relationship with other individuals.

#### Theme 1: Past trauma.

Many of the participants (N = 8) discussed incidents or traumatic events that may have triggered their anxiety. Some of these events may have happened to them personally or to people that they knew. Events ranged from near-miss accidents to knowing someone who was in or died in a motor vehicle collision to being involved in a collision themselves. Participant 10 reported an encounter with a near-miss accident that sparked her driving anxiety:

*“I was driving to a conference for work, and a tractor-trailer crossed the median into the green coming into our lane and almost hit us, but he turned just in time. So ever since then, I have not really enjoyed driving the interstate.”*

Participant 7 reported knowing someone personally involved in a traumatic accident; they stated,

*“The same year, my mom was hit by a drunk driver, and her car exploded, and the only reason she survived was because a volunteer firefighter was able to pull her out through the sunroof before it exploded. So that was all before I drove. I was always well aware that there were bad situations.”*

Participant 10 reported a similar experience with knowing someone who died from being involved in an accident.

*“So I think it [anxiety symptoms] actually started before I even got my driver’s license. I can remember it was the summer before I turned 16. And within that summer, and I guess right after that, after my birthday, I had a friend who I went to kindergarten through prior to high school. He actually died on his 16th birthday because he was in a car accident and flew through the windshield and hit a tree, and died. And I can remember telling my mom and dad, I don’t want to get my driver’s license, and the girl must have, maybe she passed away the fall before, but she also hit a tree and passed away at the driver’s seat. She was plus size, I’m plus size, and the airbag deployed, and it suffocated her. And I can just remember going, gosh, I don’t want any of that kind of stuff to happen. So I think I have always been anxious before I even truly got behind the wheel of a car.”*

#### Theme 2: Environmental factors that cause anxiety and the inability to control them.

Virtually all of the participants discussed environmental factors that caused their driving anxiety to increase. These factors included inclement weather, traffic patterns, road layout, bridges, tunnels, physical signs, time of day, other drivers, and those drivers’ behavior. Many participants felt these were things they could not necessarily control and that is what made them anxious. For example, Participant 9 reported how inclement weather exacerbates their anxiety symptoms even before they are in their vehicle:

*“Well, the first thing would be bad weather. That would be the first time on list slippery roads, because that’s where the problem originally started on an icy road. So I could start being affected by that by the night before. If the weatherman says tomorrow we’re going to have a little ice and slippery [conditions] on the road, that’s where it [anxiety] starts. It doesn’t start when I’m actually driving. It starts as somebody mentions it.”*

Participant 7 described how the time of day impacts their driving anxiety and their “in-the-moment” coping strategy they use to manage their symptoms:

*“I like to avoid when most people are on the roads, so I drive during the daylight and specifically I avoid that ‘6:00 AM to 8:00 AM’ time window or the ‘4:00 PM to 6:00 PM’ window. As long as we’re voicing that we can make, it’ll be fine.”*

#### Theme 3: Both out of vehicle and within vehicle coping strategies.

Every participant discussed their unique management strategies to deal with their driving anxiety. Some participants engaged in behaviors to reduce their anxiety while driving (i.e., within the vehicle strategies) and some performed these behaviors before they even got in the vehicle (i.e., outside the vehicle strategies). Common within vehicle strategies included gum chewing, praying, positive self-talk, stopping any distractions, or purposely engaging with distractions such as radio, podcasts, people, etc. Most participants also engaged in safety behaviors like seat belt use, checking tire air pressure, etc before their trips. Outside of the vehicle, many participants planned their route ahead of time to decrease their anxiety or engaged in general anxiety-reducing behaviors (e.g., medication, talk therapy, exercise, acupuncture, etc). Some even purposefully bought larger or safer vehicles to increase their security or even moved closer to places they needed to get to such as work. For example, Participant 2 discussed their planning process before they began their drive (e.g., out of vehicle coping strategy):

*“Normally, now I will pull up an app and look at the best way to take before I go anywhere, especially in Morgantown.”*

Similarly, Participant 4 described their route planning as a coping mechanism (e.g., out of vehicle coping strategy):

*“Yeah, so planning my route, not just using a GPS in the moment, but looking at it before, if I go to a restaurant I’m not familiar with, I either ask the friend who invited me if there’s a parking lot or I’ll look on Google Maps and actually zoom in and see if there’s parking.”*

Three sub-themes regarding coping mechanisms did emerge from the participants’ interviews as well. This included distraction, reliance on others, or avoidance of driving or aspects of driving that increase anxiety.

**Theme 3, Sub-theme 1: Distraction**

Many participants discussed trying to distract themselves or even limit distractions as a means of coping with their anxiety while driving. Many participants listened to music, purposefully sung, ate, repeated a prayer or mantra, as a means of distracting themselves from their own feelings. Some individuals purposefully limited these distractions to become hyper-focused on driving and as a way to decrease anxious thoughts. As Participant 1 explained:

*“When I’m anxious or worked up, I have to kind of distract myself mentally. So I’ll try to focus on the radio and singing or something to that effect.”*

**Theme 3, Sub-theme 2: Reliance on others**

Several participants relied on others as a coping mechanism. Some wanted a passenger along to distract them or a passenger to be there in case they could no longer drive due to their anxiety. As Participant 1 reported:

*“I’ll try to, if I have a passenger, which I always prefer to have one because I feel safer with somebody with me. If I have an issue, I can be like, okay, I can pull over, and that person can take over.”*

Similarly, Participant 6 reported always trying to have a driving companion as a coping strategy:

*“I switch with my partner. We usually try to drive together.”*

**Theme 3, Sub-theme 3: Avoidance**

Many participants avoided driving when their anxiety was high. Some even went to great lengths to avoid things that made their driving anxiety worse. Many discussed that they knew avoidance was not good for their mental health, but they did it anyway. For example, Participant 1 described taking alternate routes or avoiding driving as a coping mechanism.

*“Or even if it’s something that’s close by, relatively speaking, regionally, more than likely I won’t drive there or it takes me a long time to get there because I’ll take back roads if it is something that I do want to go to.”*

Similarly, Participant 4 stated that avoidance has been a coping strategy for them as well:

*“Another way I cope is just avoidance. I just don’t go, or I just avoid going.”*

Participant 9 describes how road conditions impact whether they drive or not:

*“However, if the roads are bad, I don’t drive at all.”*

#### Theme 4: Being out of control in an unfamiliar situation or fear of hurting self, others, or property.

Virtually all participants discussed how their driving anxiety was a result of either feeling out of control of themselves in unfamiliar situations or the fear of hurting themselves, others, or property. For example, Participant 1 expressed how these anxieties impact them:

*“And then my main one that is rational is that I don’t want to do anything if I would act erratic or freak out or do anything stupid that I would hurt somebody that was in the car with me or somebody else around me. Once again, I mean it’s more realistic, but I know it’s still a little irrational, but it’s like that bothers me.”*

Participant 3 also discussed the fear of unfamiliar situations

*“I’ve started to notice that if I’m going somewhere that I specifically have never been before or don’t know where it is, I do typically feel a little bit more anxious in those situations.”*

#### Theme 5: Honoring responsibilities, being independent, and maintaining relationships.

Even though driving anxiety impacted participants’ lives to varying degrees, all of them had different motivations to continue driving. Many discussed the lack of public transportation and not having other options to get where they needed and when they needed to. Some discussed that they did not want to lose their independence or have to always rely on others. Virtually all agreed that driving allowed them to maintain relationships with family, friends, colleagues, etc. For example, Participant 2 described the importance of remaining self-sufficient:

*“I want to be self-sufficient and I don’t want it to depend on anyone.”*

Participant 5 discussed the internal struggle of maintaining independence versus driving cessation.

*“Just a pain in the butt to otherwise not, it’s more of a pain I think to work from home because I have my son, he needs to go to school so I can work and my dog and cat would be a very big distraction. And then just depending on other people to drive you, it’s too hard. We’ve all, I’m sure had to carpool in certain situations when your car’s in the garage or something and it’s a pain. So just having that independence outweighs, unfortunately, the small anxieties.”*

Finally, Participant 3 discussed how responsibilities and social relationships were their motivations to continue driving:

*“Got to go, got to do it. I got to go to work and it has to happen. I have to go visit my family. That’s a must. These things, I just know that it’s kind of like the carrot at the end for a bunny, right? It’s an idea of there’s something good on the other side of this and there’s an obligation on the other side of this and I have to do it, I can’t not do it [stop driving].*

## Discussion

The results of this study align with the current literature regarding the outstanding impact driving anxiety can have on an individual’s daily life, as well as the factors that contribute to their anxiety and methods used to manage their symptoms [2,3]. While the initial onset of driving anxiety symptoms differed for each participant, responses related to past trauma were similar to the work by Fisher et al [2] and Taylor and Deane [10]. Witnessing a collision, concerns about road dangers, or being in a collision can all lead to the onset of driving anxiety.

While the management strategies that individuals used to combat driving anxiety were individualized, they generally fell into three categories: distraction, reliance on others, or avoidance. Participants distracted themselves from their own emotions by various means such as listening to music, practicing self-talk, praying, eating, etc. Some would eliminate all distractions and become hyper-focused on driving. Participants would often rely on others if their anxiety was perceived as high; this included having a passenger, calling someone while driving, or even letting someone else drive. Participants would also avoid driving altogether or avoid specific situations that made their driving anxiety worse. It appeared that each participant had a different battery of coping mechanisms or skills [7] they utilized following a past traumatic event. However, avoidance was a consistent strategy indicated within the sample, as well as co-dependence/reliance on others; these findings were similar to the work of Hidalgo-Munoz et al [19]. Despite their driving anxiety, participants were still internally motivated to continue to drive to honor their responsibilities, maintain independence, and social connectedness.

While continuing to drive was important to participants, the symptom management strategies mentioned could have their own repercussions. For example, previous research has shown that avoidance behaviors are associated with higher levels of anxiety [9]. It is not clear in the literature if avoidance causes anxiety or anxiety causes avoidance. While this study did not have participants rate their perceived level of anxiety, avoidance was present through the discussion of alternate routes, canceling social engagements, or depending on someone else to drive them. Also, distracted driving can be immensely dangerous; in 2022 alone, nearly 11% of all police-reported collisions were attributed to distracted driving [20]. Additionally, previous research has shown that reliance on others can impact mental health [21,22]. Thus, while these strategies may have unintended consequences on mental health and/or safety, it is possible that these would be less detrimental to participants’ well-being than not making commitments or missing social events.

Research has shown that driving is a highly valued activity that enables individuals’

independence since it is a preferred means of transportation [23]. Driving anxiety may be detrimental to individuals’ overall health and well-being, as it can significantly impact quality of life and general psychological state [4,19]. This specific anxiety has been studied over the course of many years providing ample data for professionals to use in clinical practice. However, this work adds a unique layer to this research as this study took a qualitative approach to understanding how individuals cope with their driving anxiety. While each strategy was individualized, it is important that professionals discuss with individuals options of alternate means of transportation, altering routines related to driving patterns, or initiating planning strategies before beginning their routes to help cope with driving anxiety.

### Limitations

While this study adds to the limited extant literature on driving anxiety, it is not without limitations. First, the study participants were homogenous and consisted of individuals who all identified as middle-aged, white, females with a similar demographic makeup. The limited diversity within the sample potentially reduces the generalizability of the results. Secondly, recruitment strategies were implemented in the geographic location of Morgantown, WV. This particular area has limited alternate driving options; this could be a reason why participants continue to drive despite having driving anxiety symptoms. Additionally, as these were interviews, it is possible that reporting and recall bias existed. It is possible that participants did not recall information correctly or that they provided socially acceptable responses. However, several steps were taken to minimize this. All demographic characteristics were anonymized and collected via a computer tablet and interviewees could choose their modality in which the interview was conducted (e.g., phone, Zoom/Teams, in-person).

### Implication for future research

This study serves as a foundational study to capture the qualitative responses to better understand the impact driving anxiety has on individuals. In addition to capturing the lived experiences, this study aimed to discover how individuals manage and their anxiety symptoms while driving, which was determined to be a gap in the current literature; most current studies focus on quantifying anxiety ratings and level of impact. Future studies could explore the effectiveness of anxiety management strategies related to different driving situations. The importance of honoring responsibilities, maintaining autonomy/independence, and social connectedness speaks to the importance of initiating and having individualized coping mechanisms to combat driving anxiety.
